# A Deep Learning Framework for Automated Classification and Archiving of Orthodontic Diagnostic Documents

**DOI:** 10.7759/cureus.76530

**Published:** 2024-12-28

**Authors:** Shahab Kavousinejad, Zahra Ameli-Mazandarani, Mohammad Behnaz, Asghar Ebadifar

**Affiliations:** 1 Department of Orthodontics, School of Dentistry, Shahid Beheshti University of Medical Sciences, Tehran, IRN; 2 Dentofacial Deformities Research Center, Research Institute of Dental Sciences, Shahid Beheshti University of Medical Sciences, Tehran, IRN

**Keywords:** artificial intelligence, artificial intelligence in dentistry, automation, classification, computer vision, convolutional neural networks (cnn), deep learning, orthodontics

## Abstract

Background

Orthodontic diagnostic workflows often rely on manual classification and archiving of large volumes of patient images, a process that is both time-consuming and prone to errors such as mislabeling and incomplete documentation. These challenges can compromise treatment accuracy and overall patient care. To address these issues, we propose an artificial intelligence (AI)-driven deep learning framework based on convolutional neural networks (CNNs) to automate the classification and archiving of orthodontic diagnostic images. Our AI-based framework enhances workflow efficiency and reduces human errors. This study is an initial step towards fully automating orthodontic diagnosis and treatment planning systems, specifically focusing on the automation of orthodontic diagnostic record classification using AI.

Methods

This study employed a dataset comprising 61,842 images collected from three dental clinics, distributed across 13 categories. A sequential classification approach was developed, starting with a primary model that categorized images into three main groups: extraoral, intraoral, and radiographic. Secondary models were applied within each group to perform the final classification. The proposed model, enhanced with attention modules, was trained and compared with pre-trained models such as ResNet50 (Microsoft Corporation, Redmond, Washington, United States) and InceptionV3 (Google LLC, Mountain View, California, United States). External validation was performed using 13,729 new samples to assess the artificial intelligence (AI) system’s accuracy and generalizability compared to expert assessments.

Results

The deep learning framework achieved an accuracy of 99.24% on an external validation set, demonstrating performance almost on par with human experts. Additionally, the model demonstrated significantly faster processing times compared to manual methods. Gradient-weighted class activation mapping (Grad-CAM) visualizations confirmed that the model effectively focused on clinically relevant features during classification, further supporting its clinical applicability.

Conclusion

This study introduces a deep learning framework for automating the classification and archiving of orthodontic diagnostic images. The model achieved impressive accuracy and demonstrated clinically relevant feature focus through Grad-CAM visualizations. Beyond its high accuracy, the framework offers significant improvements in processing speed, making it a viable tool for real-time applications in orthodontics. This approach not only reduces the workload in healthcare settings but also lays the foundation for future automated diagnostic and treatment planning systems in digital orthodontics.

## Introduction

Dental informatics, as an emerging field, harnesses information technology to improve clinical outcomes by digitizing diagnostic and treatment workflows. This digital transformation not only streamlines processes, saving time for both dentists and patients, but also minimizes stress by providing more efficient and effective oral healthcare [1]. While there is no definitive consensus in the scientific literature on the minimum documentation required for orthodontic diagnosis and treatment planning [2-7], a typical set of intraoral and extraoral photographs, along with specific radiographs, is generally considered essential for standard evaluation. These typically include panoramic, lateral cephalometric, and sometimes posteroanterior cephalometric images [8].

Contemporary dental practices increasingly rely on digital systems, such as electronic health records (EHRs) and patient management systems, to organize and store diagnostic records [9]. This digital approach provides dentists with easy access to past records, enabling them to conveniently refer to these documents for ongoing patient monitoring or future needs [10]. However, traditional methods of data storage in dentistry relied on manual image labeling, which was both time-consuming and prone to errors, especially when managing large datasets [11,12].

The advent of artificial intelligence (AI), particularly deep learning, promises to revolutionize this process by automating image classification and archiving. AI empowers dental practices to better organize existing image data and streamline the archiving of new images [13]. AI-powered algorithms are proving invaluable for analyzing dental images, including radiographs, intraoral scans, and panoramic images, to identify and classify dental restorations, caries, bony lesions, and maxillofacial abnormalities [14-17]. For instance, deep learning models have shown improvements in segmenting features in dental periapical radiographs, such as carious lesions, crowns, dental pulp, and root canal fillings [18]. Deep learning, a subset of machine learning, uses multi-layered artificial neural networks to extract complex patterns from data [19]. Convolutional neural networks (CNNs), a prominent deep learning architecture, excel in image analysis tasks [20]. CNNs automatically learn hierarchical visual features from raw image pixels through deep layers and pooling operations, making them particularly effective for visual information interpretation in computer vision tasks [21]. Recent studies have demonstrated the effectiveness of CNNs in dental applications, such as detecting cephalometric landmarks [22], facial analysis [12], orthodontic treatment planning [12,23], and diagnosing dental plaque [24] and caries [25].

Our literature review shows that few studies have comprehensively classified orthodontic image sets, including intraoral, extraoral, and radiographic images [26-28]. The present study hypothesizes that deep learning algorithms will outperform traditional image classification methods in categorizing orthodontic images. Previous studies have been limited by single-center datasets [27,28], exclusion of radiographic images [27], and reliance on simplistic features like grayscale or aspect ratio [26], which may result in unreliable outcomes in real-world applications. This research aims to address these limitations by designing a custom CNN architecture based on attention mechanisms, along with advanced preprocessing techniques, and comparing it with existing models. The models will then be trained and validated. Their performance will be evaluated on test data. Additionally, the models will undergo external validation and be compared with expert assessments. This research is a starting point for creating an AI-based system to help with automatic diagnosis and treatment planning in orthodontics and orthognathic surgery. The first step is to design a system that can classify orthodontic images and documents using computer vision and CNNs. In future studies, this ability can be expanded to detect dental issues, identify malocclusions, and measure specific features. For example, if the system identifies an upper jaw image, it can estimate crowding or arch width in the next steps. This study focuses on training the system to recognize and categorize orthodontic images, establishing a foundation for future automated treatment workflows.

## Materials and methods

Data collection and dataset preparation

**Figure 1:**
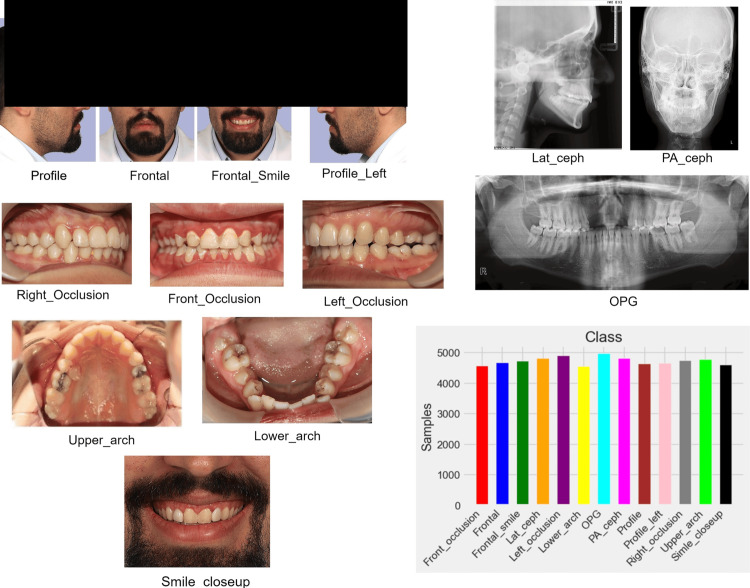
Orthodontic diagnostic records and the quantity of each type included in this study. Lat-Ceph: lateral cephalometry; OPG: orthopantomograph; PA-Ceph: posteroanterior cephalometry

To develop an accurate model for the automated classification of orthodontic diagnostic documents, a dataset was compiled from an archive of patient records ranging from seven to 40 years of age. The study was conducted at the School of Dentistry, Shahid Beheshti University of Medical Sciences, Tehran, Iran. The dataset included a diverse range of records from three dental clinics in Tehran, Iran, ensuring a balanced representation of various populations and demographics. Privacy and patient rights were prioritized during data collection by removing all personally identifiable information. The dataset consisted of 61,842 images, including intraoral images, both with and without orthodontic appliances, as well as extraoral and radiographic images, ensuring a diverse range of diagnostic documents (Figure 1). These images were used to train the AI models and evaluate their generalization across different data samples. All identifiable patient information, including headers and Digital Imaging and Communications in Medicine (DICOM) data, was removed from radiographic images. The anonymized images were then converted to JPEG format and compiled into a single dataset. Inclusion criteria for the dataset required that the images represented various orthodontic diagnostic categories, including intraoral images with or without orthodontic appliances, extraoral images, and radiographic images, while complying with privacy regulations through the removal of all personally identifiable information, such as DICOM headers and patient details. Exclusion criteria involved the removal of images that were of very poor quality, contained artifacts or had very low resolution, as these could impair model training. The study was approved by the Ethics Committee of the Faculty of Dentistry at Shahid Beheshti University of Medical Sciences (code IR.SBMU.DRC.REC.1403.025). The study used an archive of past patient records, and informed consent was waived due to the inability to contact the patients directly. All personal identifying information was removed to ensure anonymity.

The dataset was manually classified into 13 distinct categories: front-occlusion, frontal, frontal-smile, lateral cephalometry (Lat-Ceph), left-occlusion, lower-arch, orthopantomograph (OPG), posteroanterior cephalometry (PA-Ceph), profile, profile-left, right-occlusion, upper-arch, and smile-closeup (Figure 1). Each image was initially annotated by an orthodontic expert, and the classifications were subsequently reviewed and verified by a second expert to ensure consistency and accuracy.

Data preprocessing

**Figure 2:**
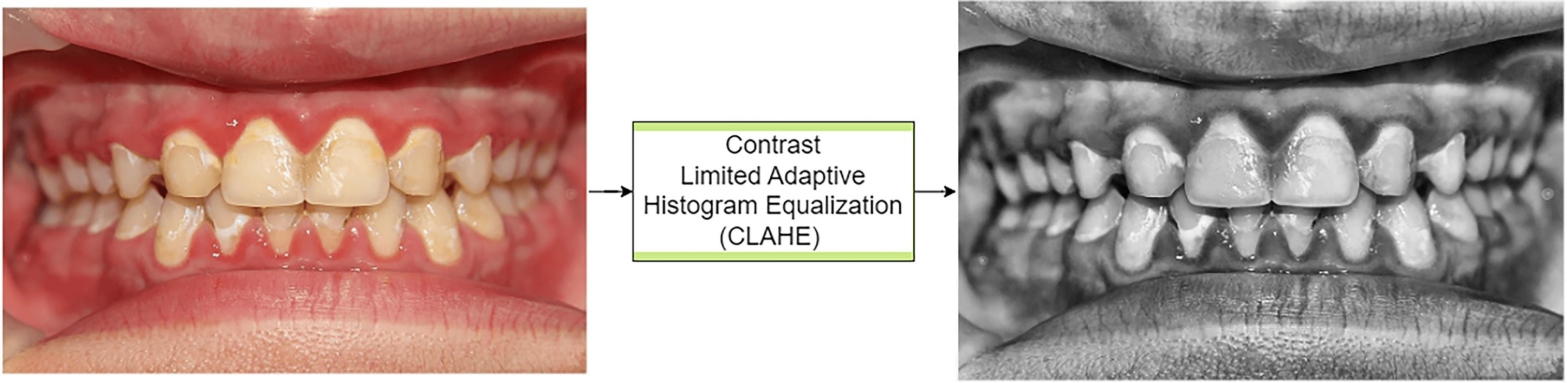
The combined application of CLAHE and grayscale conversion enhances image quality by improving contrast and reducing color complexity. CLAHE: contrast limited adaptive histogram equalization

To ensure high-quality and consistent input images during training, several preprocessing steps were applied. The contrast-limited adaptive histogram equalization (CLAHE) was used to enhance image contrast and emphasize important details (Figure 2). All images were converted to grayscale to maintain consistency across input formats. They were resized to 128x128 pixels to standardize the input dimensions, ensuring uniformity for the model's processing. After resizing, the images were normalized to a range of 0 to 1, rescaling pixel values from the typical 0-255 range to improve model stability and accelerate training. To increase dataset diversity, data augmentation techniques, including random rotations (up to 5 degrees), were applied, generating additional images with varied orientations. These augmented images were used in batches during each training epoch, helping the model improve by exposing it to a broader set of image variations.

Proposed model architecture and technical aspects

**Figure 3:**
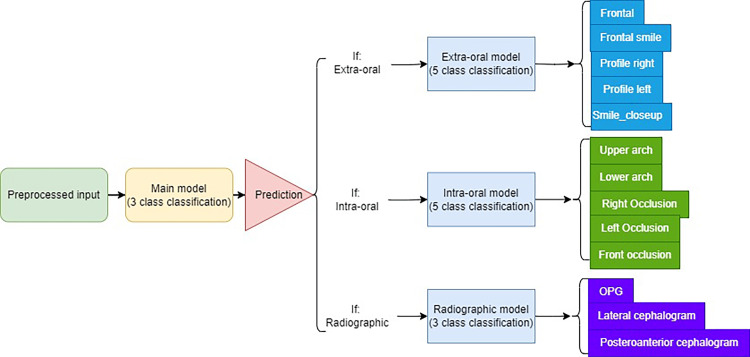
Overview of the multi-model classification framework. In this study, four models were developed. The primary model was designed to first classify the image as either extraoral, intraoral, or radiographic. Based on the output of the primary model, the image was then passed on to the appropriate secondary model, each responsible for performing the final classification specific to its image type. Lat-Ceph: lateral cephalometry; OPG: orthopantomograph; PA-Ceph: posteroanterior cephalometry

**Figure 4:**
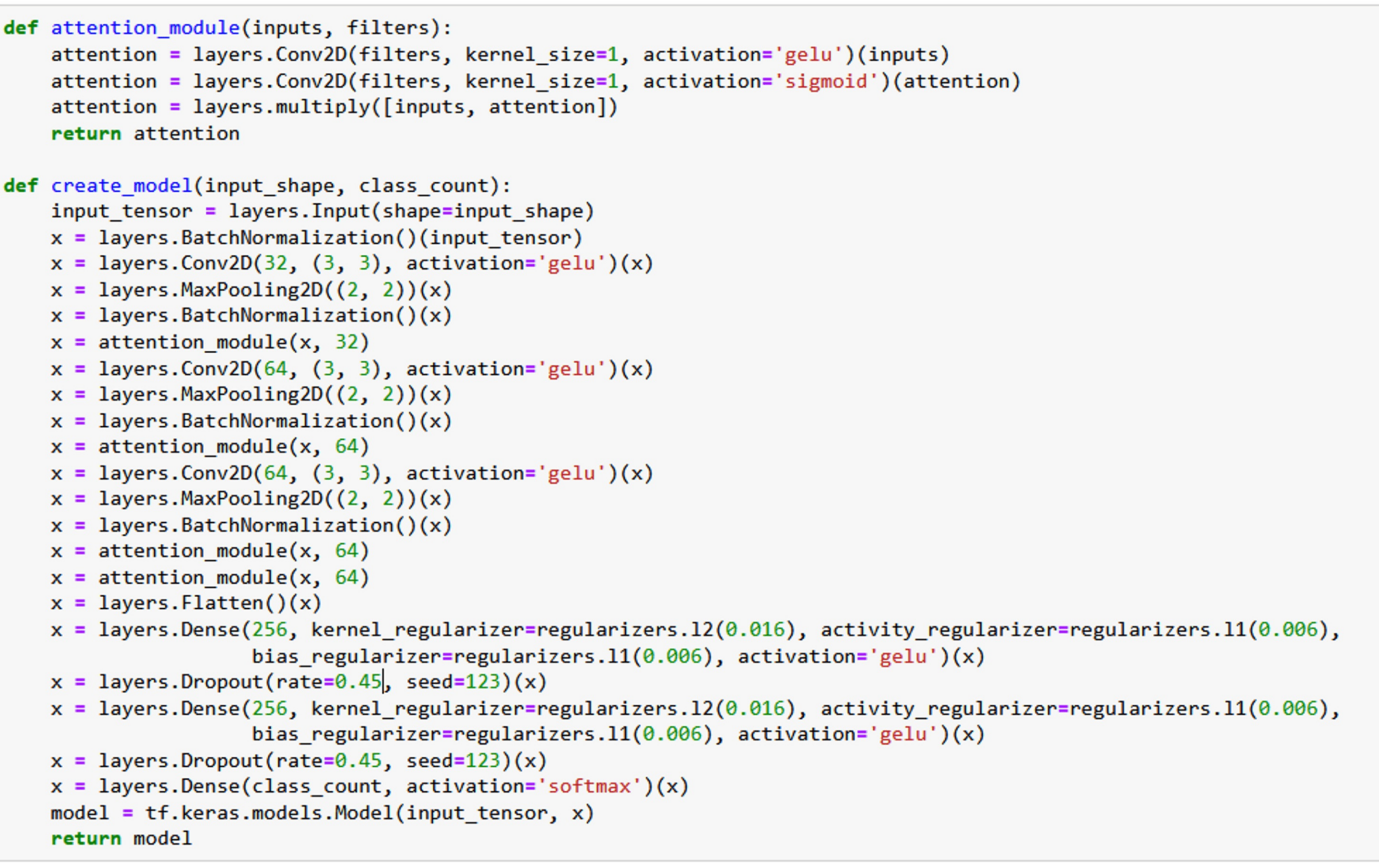
ACNN architecture incorporating attention mechanisms. This implementation demonstrates the model architecture using the Python programming language, with libraries such as TensorFlow 2.6 and Keras 2.6 in a Python 3.9 environment. ACNN: attention-based convolutional neural network; GELU: Gaussian error linear unit; Conv2D: 2D convolutional layer

In this study, a pipeline consisting of four models was developed to classify different types of images (Figure 3). The initial goal was to classify the images into three main classes: extraoral, intraoral, and radiographic. A primary model (main model) was first trained to classify the images into one of these three categories. Based on the output of the primary model, the image was then routed to the appropriate secondary model, each responsible for performing the final classification specific to its respective image type. Extraoral images were further classified into one of five specific classes by the extraoral model, intraoral images were classified into one of five classes by the intraoral model, and radiographic images were classified into one of three classes by the radiographic model. Each model employed a unique architecture and was trained independently on its respective dataset, ensuring optimal performance at each stage of the classification pipeline. This sequential approach ensured efficient processing, passing each image to the correct model for accurate classification. The architecture of each of the four models in the pipeline is depicted in Figure 4.

The proposed attention-based convolutional neural network (ACNN) architecture for image classification integrated attention modules, the main architecture, and dense layers (Figure 4). The attention module captured spatial information, allowing the model to focus on salient features, which enhanced classification accuracy. The main architecture comprised convolutional layers for feature extraction and downsampling, while dense layers computed the final class probabilities. The architecture included custom CNN layers that utilized convolutional operations with kernel filters and Gaussian error linear unit (GELU) activation. Attention modules further enhanced feature emphasis by employing additional convolutional layers with GELU and sigmoid activations, multiplying inputs by attention weights to prioritize relevant information. A fully connected layer with GELU activation introduced non-linearity, enabling the model to learn complex patterns more effectively than traditional activation functions. To combat overfitting, dropout was applied at a rate of 0.45, randomly deactivating neurons during training, while weight regularization techniques (L1 = 0.006, L2 = 0.016) constrained weights, preventing excessive model complexity. The convolutional layers featured varying filter sizes: the initial layers had 32 filters (3x3), while the subsequent layers had 32 and 64 filters, respectively. GELU activation was predominantly used, with attention modules incorporating both GELU and sigmoid activations. The model employed the Adamax optimizer, selected for its robustness in handling sparse gradients, making it suitable for this classification task. A categorical cross-entropy loss function was utilized for multi-class classification. The architecture and hyperparameters underwent fine-tuning through a systematic search process, including grid search, to optimize performance based on validation metrics. Training was performed with a batch size of 32 for up to 100 epochs, implementing early stopping based on validation loss, which halted training when no significant improvement was observed over a set number of epochs. This methodology ensured effective generalization and mitigated overfitting. Table 1 shows the architecture of the ACNN.

**Table 1:** ACNN architecture incorporating attention mechanisms and residual connections. In this architecture, the input layer receives batches of images with dimensions of 128x128 pixels and a single color channel (grayscale). BatchNormalization layers are applied to normalize the data, followed by Conv2D layers with 32 and 64 filters for feature extraction. MaxPooling2D layers reduce the image dimensions, while Attention-Module layers help the model focus on the most important features. The data is then flattened using the Flatten layer, and Dense layers with 256 units combine the extracted features in a fully connected manner. Finally, a Dropout layer is applied to prevent overfitting, and the output layer, with a number of units corresponding to the number of classes, makes the final prediction. ACNN: attention-based convolutional neural network; GELU: Gaussian error linear unit; Conv2D: 2D convolutional layer

Layer	Type	Output Shape	Activation	Notes
Input	Input Layer	(batch_size, 128, 128, 1)	-	-
Conv2D (32 filters)	Convolution	(batch_size, 126, 126, 32)	GELU	Kernel size: 3x3
MaxPooling2D	Pooling	(batch_size, 63, 63, 32)	-	Pool size: 2x2
BatchNormalization	BatchNormalization	(batch_size, 63, 63, 32)	-	Normalizes previous output
Attention-Module (32 filters)	Custom Attention	(batch_size, 63, 63, 32)	-	Uses Conv2D with GELU and sigmoid activation
Conv2D (64 filters)	Convolution	(batch_size, 61, 61, 64)	GELU	Kernel size: 3x3
MaxPooling2D	Pooling	(batch_size, 30, 30, 64)	-	Pool size: 2x2
BatchNormalization	BatchNormalization	(batch_size, 30, 30, 64)	-	Normalizes previous output
Attention-Module (64 filters)	Custom Attention	(batch_size, 30, 30, 64)	-	Applies attention mechanism
Conv2D (64 filters)	Convolution	(batch_size, 28, 28, 64)	GELU	Kernel size: 3x3
MaxPooling2D	Pooling	(batch_size, 14, 14, 64)	-	Pool size: 2x2
BatchNormalization	BatchNormalization	(batch_size, 14, 14, 64)	-	Normalizes previous output
Attention-Module (64 filters)	Custom Attention	(batch_size, 14, 14, 64)	-	Applies attention mechanism
Attention-Module (64 filters)	Custom Attention	(batch_size, 14, 14, 64)	-	Further attention layers
Flatten	Flatten	(batch_size, 12544)	-	Converts 2D features to 1D
Dense (256 units)	Dense	(batch_size, 256)	GELU	Regularization applied
Dropout (0.45)	Dropout	(batch_size, 256)	-	Dropout for regularization
Dense (256 units)	Dense	(batch_size, 256)	GELU	Regularization applied
Dropout (0.45)	Dropout	(batch_size, 256)	-	Dropout for regularization
Dense (class_count)	Dense	(batch_size, class_count)	Softmax	Final output layer for classification

To find the optimal hyperparameters, a comprehensive grid search was conducted over a wide range of hyperparameters. The learning rate was tested at values of 0.001, 0.0005, and 0.0001, while the optimizers tested included Adam, Adamax, and RMSprop. The number of filters in the convolutional layers varied between 32, 64, and 128 for the first layer and 64, 128, and 256 for subsequent layers. Dropout rates were explored from 0.2 to 0.5 in increments of 0.05. Weight regularization included both L1 and L2 values, tested within the range of 0.001 to 0.02 for L1 and 0.001 to 0.02 for L2, in increments of 0.001. The batch size varied from 16 to 128. Kernel sizes were evaluated at 3x3, 5x5, and 7x7, and activation functions like GELU and rectified linear unit (ReLU) were tested. The grid search process followed a systematic approach, where each combination of hyperparameters was evaluated independently to identify the optimal set for the model. A five-fold cross-validation technique was applied to ensure the robustness of the evaluation, allowing for a more reliable measure of performance and generalization. The process involved iterating over all possible combinations of the selected hyperparameters, training the model for each configuration, and assessing its performance on a validation set.

In this study, four different models were trained: the main model with the custom ACNN architecture (main-ACNN), the intraoral model (IO-ACNN), the extraoral model (EO-ACNN), and the radiographic model (Ra-ACNN). These models differed in their training parameters, hyperparameters, and final classification, but their core architectures were similar, with the exception of the final classification layer, as shown in Figure 4.

Model training and evaluation

**Figure 5:**
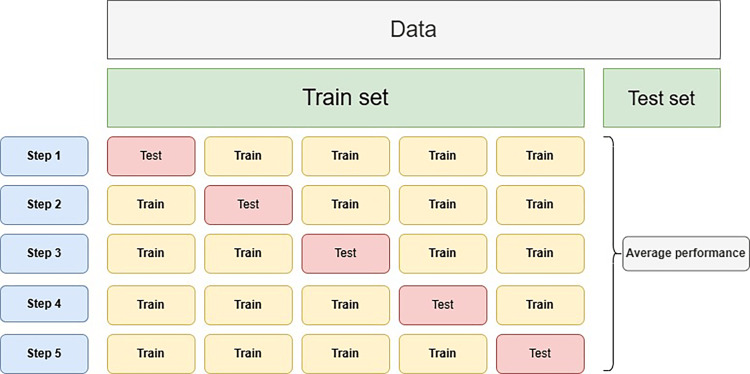
Evaluation of the model using a five-fold cross-validation approach.

The models were trained using a five-fold cross-validation approach (Figure 5), where the dataset was divided into five subsets. The model was trained and evaluated five times, each time using a different fold as the validation set. This method provides a robust estimate of performance by averaging results across iterations. Hyperparameter tuning was conducted using grid search, and to avoid bias and overfitting, a separate final test dataset was employed. After confirming satisfactory performance through cross-validation, we conducted a single evaluation on the test dataset without retuning the models.

The project was implemented in a Python 3.9 environment, utilizing TensorFlow 2.6 and Keras 2.6, and executed on an NVIDIA GeForce RTX 3090 GPU. To compare ACNN's performance, we also trained advanced models, including ResNet50 (Microsoft Corporation, Redmond, Washington, United States) and InceptionV3 (Google LLC, Mountain View, California, United States), under identical conditions. The early stopping method was employed to prevent overfitting. A separate test set, comprised of images from a different orthodontic center, ensured diversity in evaluating the models' generalization capabilities.

Model performance on the test data was assessed using various evaluation metrics, including accuracy, precision, recall, F1 score, receiver operating characteristic (ROC), and loss. Recall measures the proportion of true positives (TP) among all actual positives (TP + FN), indicating how effectively the model identifies positive instances. Precision indicates the proportion of true positives among all predicted positives (TP + FP), reflecting the accuracy of the model's positive predictions. The F1 score is the harmonic mean of precision and recall, providing a balance between the two. Accuracy represents the proportion of correct predictions (both true positives and true negatives) out of all instances (TP + TN + FP + FN). TP refers to correctly predicted positive instances, true negative (TN) refers to correctly predicted negative instances, false positive (FP) refers to incorrectly predicted positive instances, and false negative (FN) refers to incorrectly predicted negative instances.

$\text{Accuracy} = \frac{\text{TP} + \text{TN}}{\text{TP} + \text{TN} + \text{FP} + \text{FN}}$ 

$\text{Precision} = \frac{\text{TP}}{\text{TP} + \text{FP}}$ 

$\text{Recall} = \frac{\text{TP}}{\text{TP} + \text{FN}}$ 

$\text{F1} = \frac{2 \cdot \text{Precision} \cdot \text{Recall}}{\text{Precision} + \text{Recall}}$

To gain insights into the model's decision-making, we generated gradient-weighted class activation mapping (Grad-CAM) images based on the last convolutional layer of each model. Grad-CAM visualizes the regions most relevant to a model's prediction by computing the gradients of the target class with respect to the convolutional layers. It generates heatmaps that highlight the areas of the input image that had the most influence on the final classification. The speed and accuracy of diagnostic document classification were compared between the models and an expert using 20 new patient documents. The models classified these documents, and their outputs and classification times were recorded. The expert's classification times and document classifications were also measured using a timer. Statistical tests were conducted to assess significant differences in classification times between the AI framework and the expert. The Shapiro-Wilk test was used to assess the normality of the classification times for both groups. In case the data was not normally distributed, the Wilcoxon signed-rank test was used. Data analysis was performed using IBM SPSS Statistics for Windows, Version 28 (Released 2021; IBM Corp., Armonk, New York, United States)

Model deployment and external validation

After the final testing, the models were saved as h5 files. A Windows application was developed using Visual Studio 2019 and programmed in C# programming language to automatically classify images and perform external validation. The application receives images and, based on the framework designed in this study, initially passes them to the primary model. Then, based on the predictions from the primary model, the images are passed to the relevant models for final classification. Following initial debugging, 13,729 new images, unrelated to the training and testing datasets, were processed. These images were collected from the School of Dentistry at Shahid Beheshti University of Medical Sciences in Tehran, Iran. The images were randomly selected from a diverse set of patient records and consolidated into a single folder for processing. The software sequentially scanned each image, classified them based on the model's predictions, and stored them in the corresponding folders (by class name) (Video 1). The classification and archiving process was fully automated, with filenames generated to include patient details, such as the patient ID, for future reference. After the automatic classification, the folders were reviewed by an orthodontist who evaluated the accuracy of the classifications. The correctly and incorrectly classified cases were recorded for each class, and a confusion matrix was generated to evaluate the model's performance. Specifically, the percentage of misclassified cases in each class were noted. The confusion matrix, based on the orthodontist's assessment, provided the metrics of accuracy, sensitivity, specificity, and error rate, offering a detailed evaluation of the model's performance. This external validation, conducted using real-world data outside the training and testing datasets, provided a thorough assessment of the model’s ability to classify images accurately under practical conditions.

**Video 1:** A Windows application developed for the automated classification and archiving of orthodontic diagnostic images, leveraging an AI framework specifically designed in this study. Lat-Ceph: lateral cephalometry; OPG: orthopantomograph; PA-Ceph: posteroanterior cephalometry; AI: artificial intelligence

## Results

In this study, three different models were compared for each classification task. The custom model ACNN was evaluated against the ResNet50 and InceptionV3 models. Table 2 presents the evaluation metrics for the model on the test data. ResNet50 excelled in extraoral classification, while ACNN outperformed others in the main, intraoral, and radiographic models, showing lower loss values.

**Table 2:** Comparison of evaluation metrics across various models for performing four distinct diagnostic tasks. ACNN: attention-based convolutional neural network

Model	Loss (Categorical Cross Entropy)	Recall	F1 Score	Precision	Accuracy	Description	Test size (n)
Main-ResNet50	0.272	1.00	1.00	1.00	1.00	Main model (3 classes classification)	394
Main-InceptionV3	0.356	1.00	1.00	1.00	1.00	Main model (3 classes classification)	394
Main-ACNN	0.103	1.00	1.00	1.00	1.00	Main model (3 classes classification)	394
EO-ResNet50	0.020	1.00	1.00	1.00	1.00	Extraoral model (5 classes classification)	137
EO-InceptionV3	0.710	0.96	0.95	0.95	0.95	Extraoral model (5 classes classification)	137
EO-ACNN	0.122	0.98	0.98	0.98	0.98	Extraoral model (5 classes classification)	137
IO-ResNet50	0.040	0.99	0.99	0.99	0.99	Intraoral model (5 classes classification)	153
IO-InceptionV3	0.821	0.98	0.98	0.98	0.98	Intraoral model (5 classes classification)	153
IO-ACNN	0.009	1.00	1.00	1.00	1.00	Intraoral model (5 classes classification)	153
Ra-ResNet50	0.073	0.99	0.99	0.99	0.99	Radiographic model (3 classes classification)	133
Ra-InceptionV3	0.065	0.99	0.98	0.98	0.98	Radiographic model (3 classes classification)	133
Ra-ACNN	0.041	0.99	0.99	0.99	0.99	Radiographic model (3 classes classification)	133

**Figure 6:**
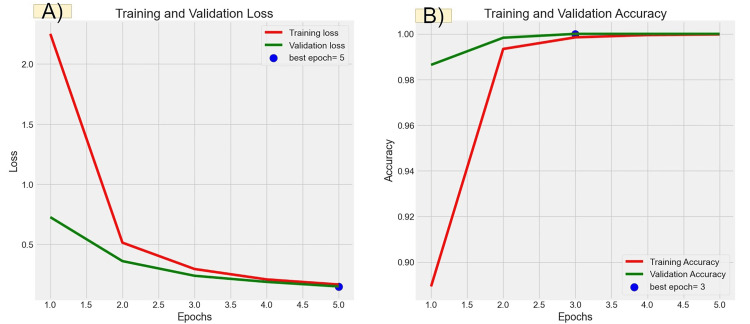
Learning curves over epochs for the main model (Main-ACNN). Panel (A) shows the learning curve for loss over epochs. The x-axis represents the number of epochs, and the y-axis shows the loss value. A declining loss curve signifies that the model is improving its performance, as it is minimizing errors. A smooth, steadily decreasing curve indicates good learning, while fluctuations could suggest instability in the training process. Panel (B) illustrates the learning curve for accuracy over epochs. The x-axis again represents the epochs, while the y-axis corresponds to the accuracy percentage. An upward trajectory in the curve indicates that the model is becoming more accurate over time. ACNN: attention-based convolutional neural network

**Figure 7:**
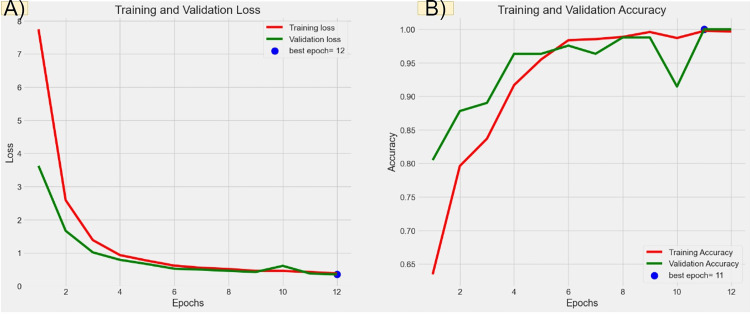
Learning curves over epochs for the best extraoral model, ResNet50. Panel (A) shows the learning curve for loss over epochs. The x-axis represents the number of epochs, and the y-axis shows the loss value. A declining loss curve signifies that the model is improving its performance, as it is minimizing errors. A smooth, steadily decreasing curve indicates good learning, while fluctuations could suggest instability in the training process. Panel (B) illustrates the learning curve for accuracy over epochs. The x-axis again represents the epochs, while the y-axis corresponds to the accuracy percentage. An upward trajectory in the curve indicates that the model is becoming more accurate over time.

**Figure 8:**
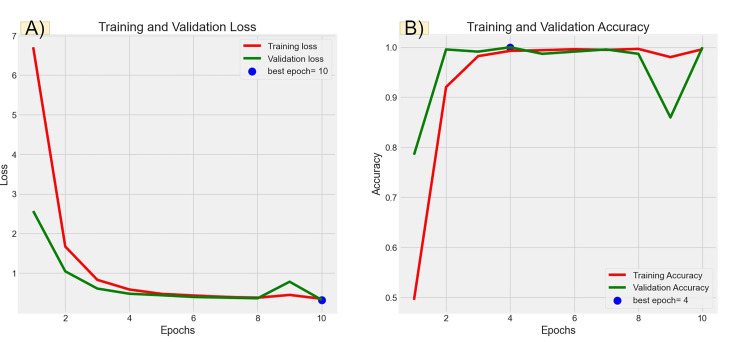
Learning curves over epochs for the best intraoral model (IO-ACNN). Panel (A) shows the learning curve for loss over epochs. The x-axis represents the number of epochs, and the y-axis shows the loss value. A declining loss curve signifies that the model is improving its performance, as it is minimizing errors. A smooth, steadily decreasing curve indicates good learning, while fluctuations could suggest instability in the training process. Panel (B) illustrates the learning curve for accuracy over epochs. The x-axis again represents the epochs, while the y-axis corresponds to the accuracy percentage. An upward trajectory in the curve indicates that the model is becoming more accurate over time. ACNN: attention-based convolutional neural network

**Figure 9:**
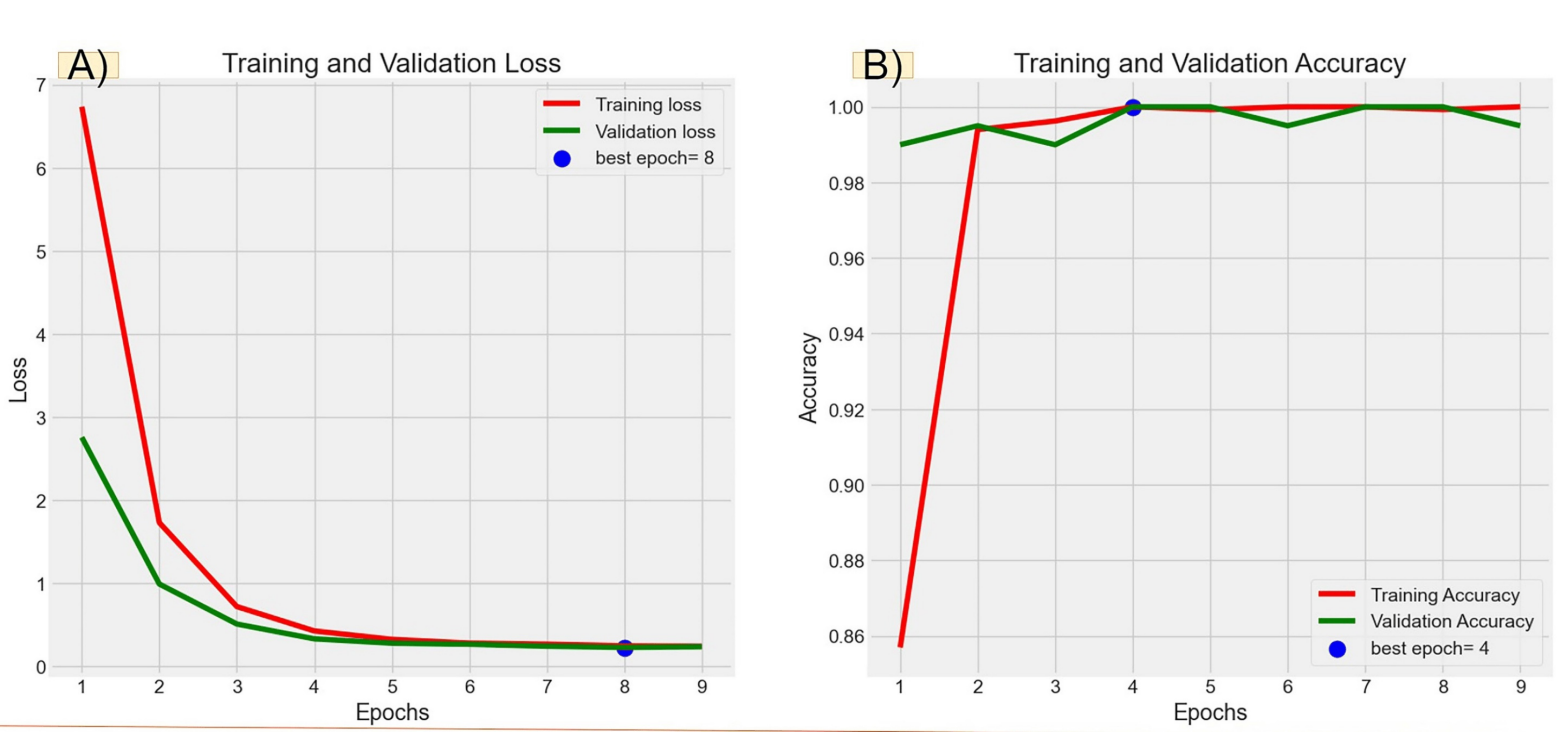
Learning curves over epochs for the best radiographic model (Ra-ACNN). Panel (A) shows the learning curve for loss over epochs. The x-axis represents the number of epochs, and the y-axis shows the loss value. A declining loss curve signifies that the model is improving its performance, as it is minimizing errors. A smooth, steadily decreasing curve indicates good learning, while fluctuations could suggest instability in the training process. Panel (B) illustrates the learning curve for accuracy over epochs. The x-axis again represents the epochs, while the y-axis corresponds to the accuracy percentage. An upward trajectory in the curve indicates that the model is becoming more accurate over time. ACNN: attention-based convolutional neural network

**Figure 10:**
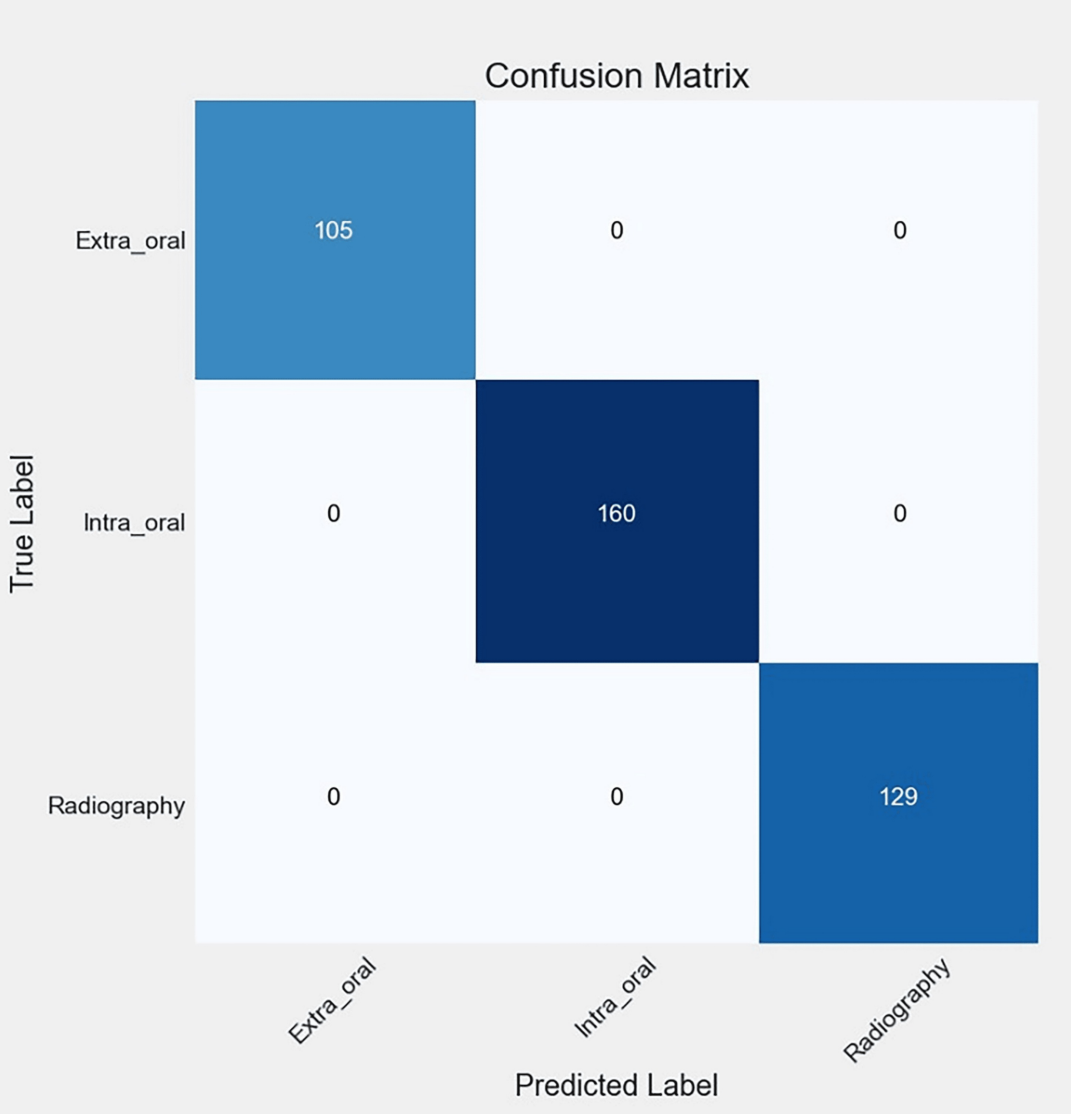
The confusion matrix of the main model, reflecting the performance of the best model (Main-ACNN) on the test data. The diagonal entries represent correct predictions, while the off-diagonal entries correspond to misclassifications. ACNN: attention-based convolutional neural network

**Figure 11:**
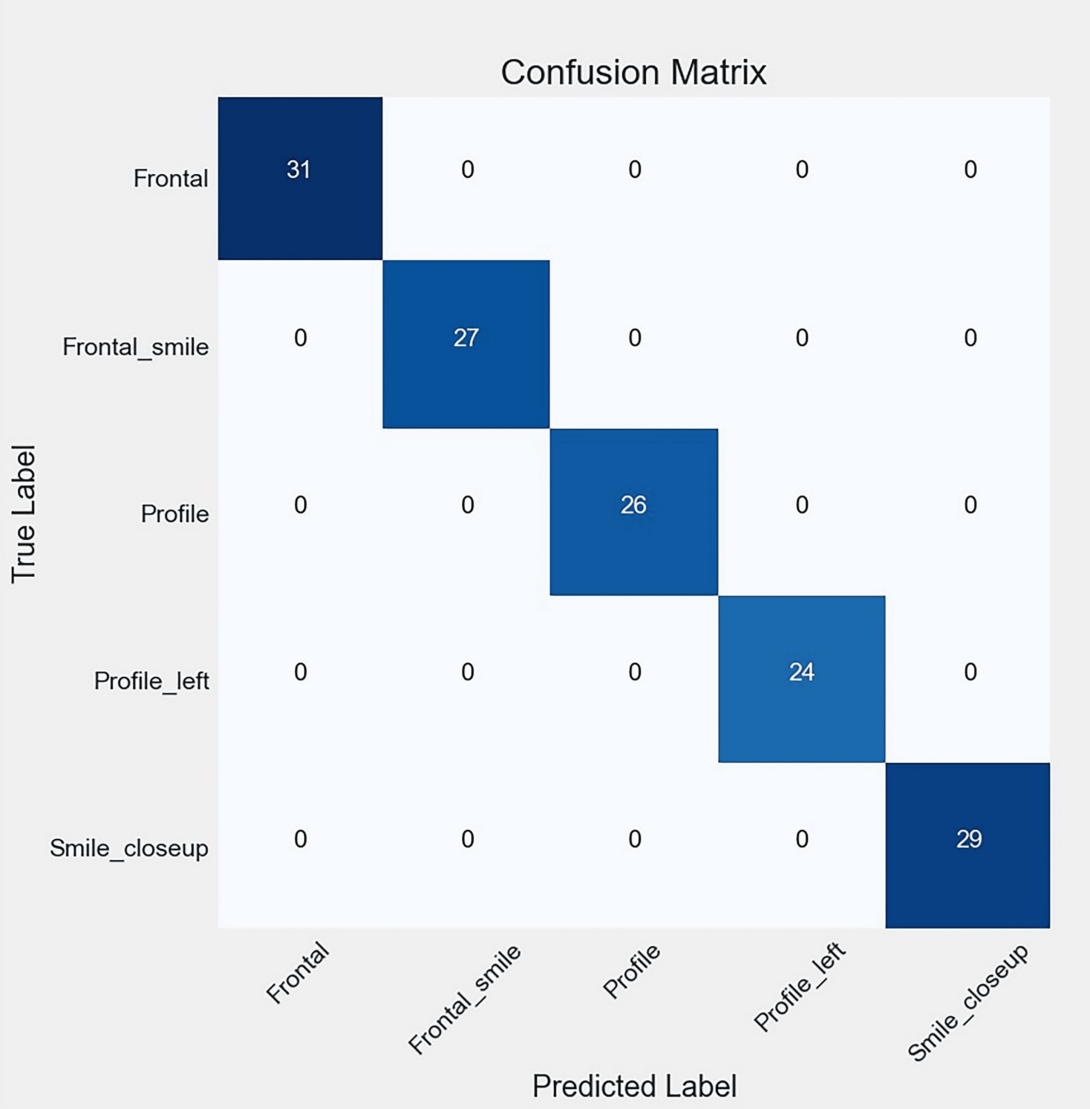
The confusion matrix of the extraoral model, reflecting the performance of the best model (EO-Resnet50) on the test data. The diagonal entries represent correct predictions, while the off-diagonal entries correspond to misclassifications.

**Figure 12:**
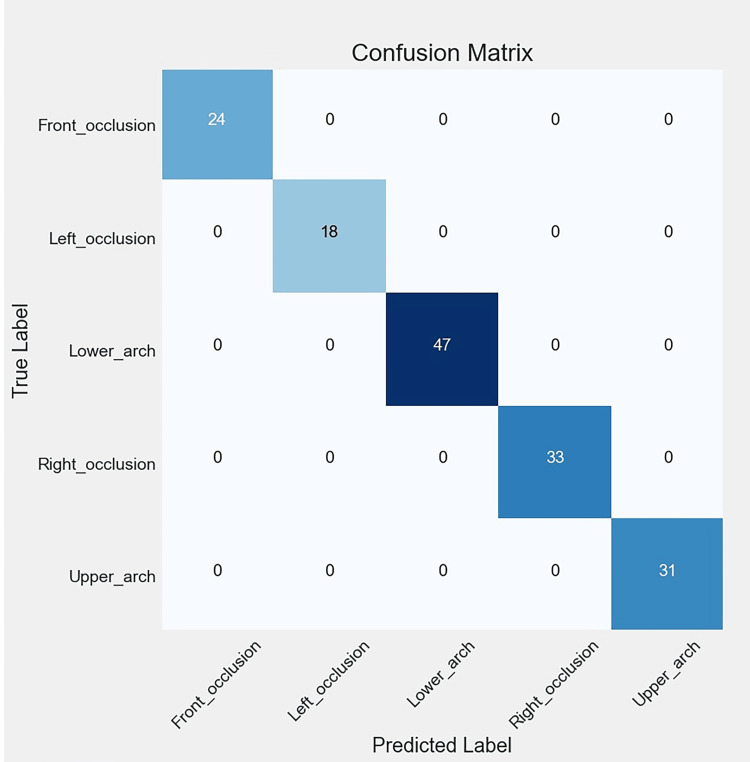
The confusion matrix of the intraoral model, reflecting the performance of the best model (IO-ACNN) on the test data. The diagonal entries represent correct predictions, while the off-diagonal entries correspond to misclassifications. ACNN: attention-based convolutional neural network

**Figure 13:**
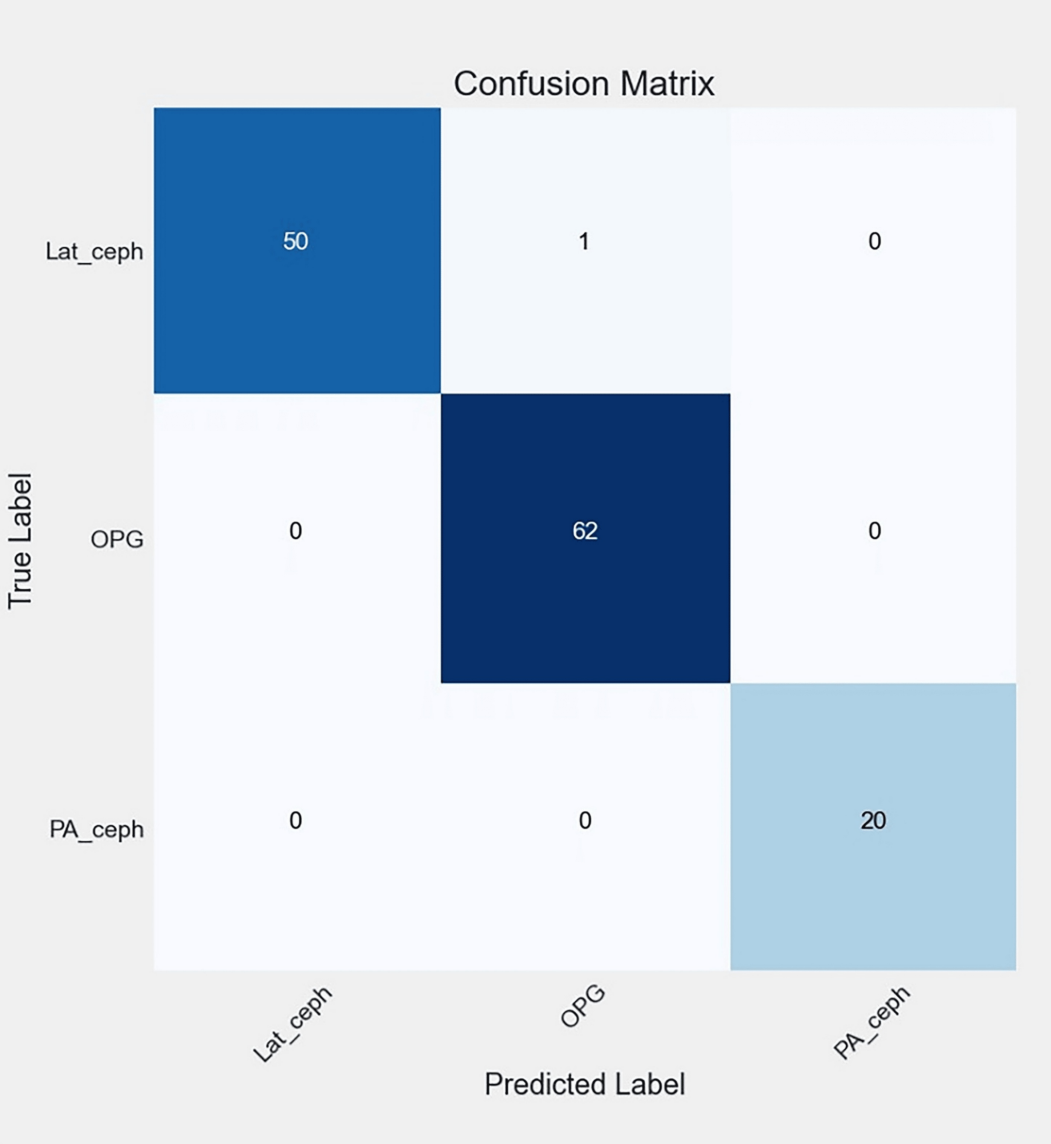
The confusion matrix of the radiographic model, reflecting the performance of the best model (Ra-ACNN) on the test data. The diagonal entries represent correct predictions, while the off-diagonal entries correspond to misclassifications. Lat-Ceph: lateral cephalometry; OPG: orthopantomograph; PA-Ceph: posteroanterior cephalometry; ACNN: attention-based convolutional neural network

**Figure 14:**
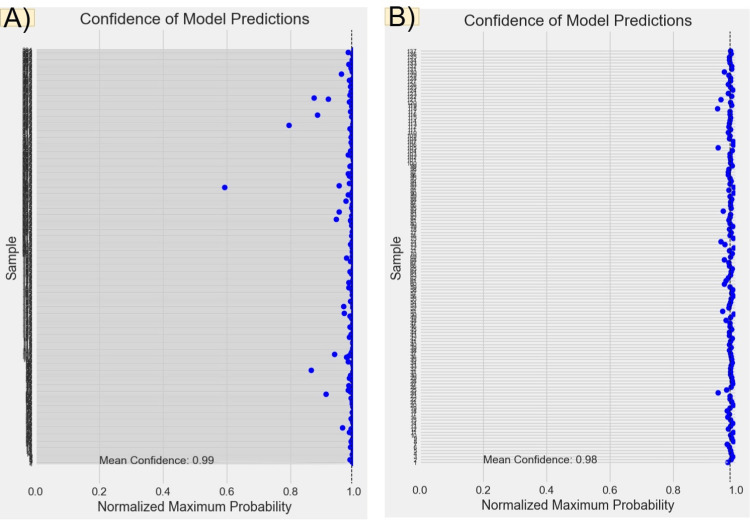
Predicted probabilities and confidence levels for main and extraoral models. Panel (A) presents the predicted probabilities for each test sample from the main model. The x-axis represents the normalized maximum probability values, while the y-axis corresponds to the test samples. This panel reflects the main model's confidence in its predictions. Panel (B) shows the predicted probabilities for each test sample from the extraoral model.

**Figure 15:**
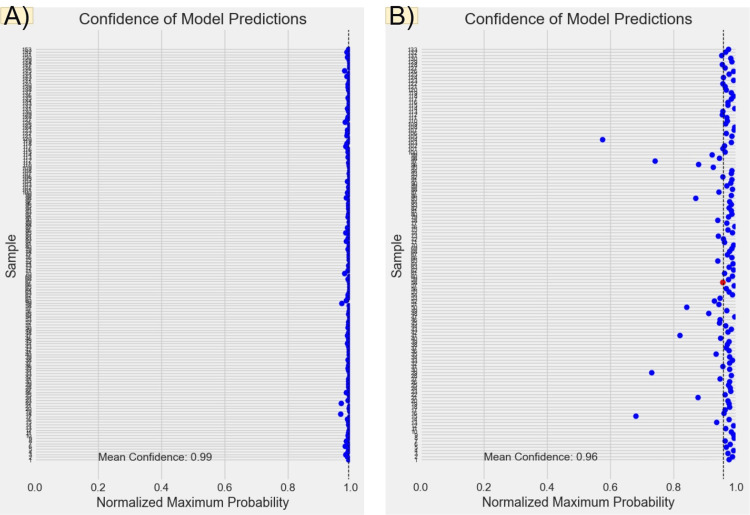
Predicted probabilities and confidence levels for inraoral and radiographic models. Panel (A) presents the predicted probabilities for each test sample from the intraoral model. The x-axis represents the normalized maximum probability values, while the y-axis corresponds to the test samples. This panel reflects the main model's confidence in its predictions. Panel (B) shows the predicted probabilities for each test sample from the radiographic model.

**Figure 16:**
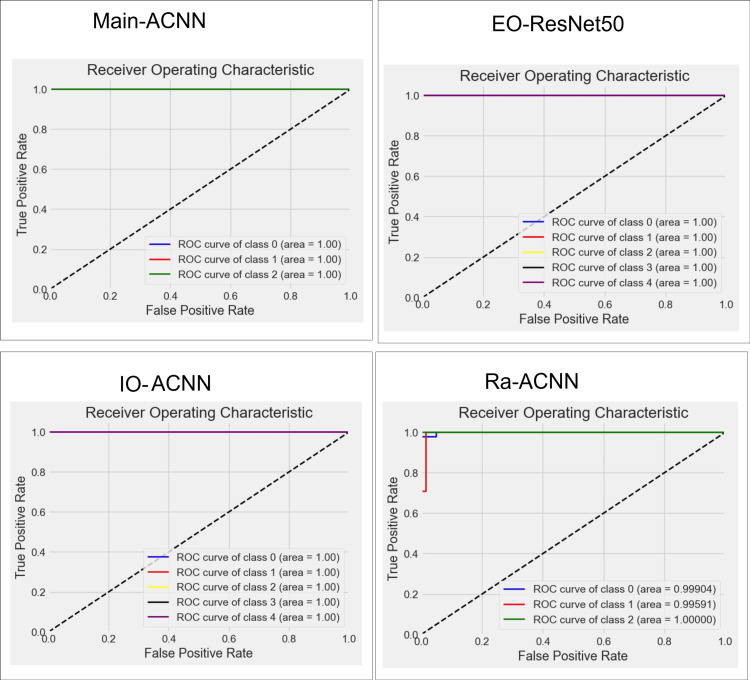
ROC curves for the best models: main, intraoral, extraoral, and radiographic. The panel illustrates the ROC curves for the best-performing models, including main, intraoral, extraoral, and radiographic, and highlights their discriminative capabilities. A curve that rises sharply toward the upper left corner indicates strong discriminative ability, while a curve that follows the diagonal suggests poor performance. The area under the curve (AUC) is a summary statistic of the ROC curve, where values close to 1.0 indicate excellent performance. ROC: receiver operating characteristic; ACNN: attention-based convolutional neural network; IO-ACNN: intraoral model; EO-ACNN: extraoral model; Ra-ACNN: radiographic model

**Figure 17:**
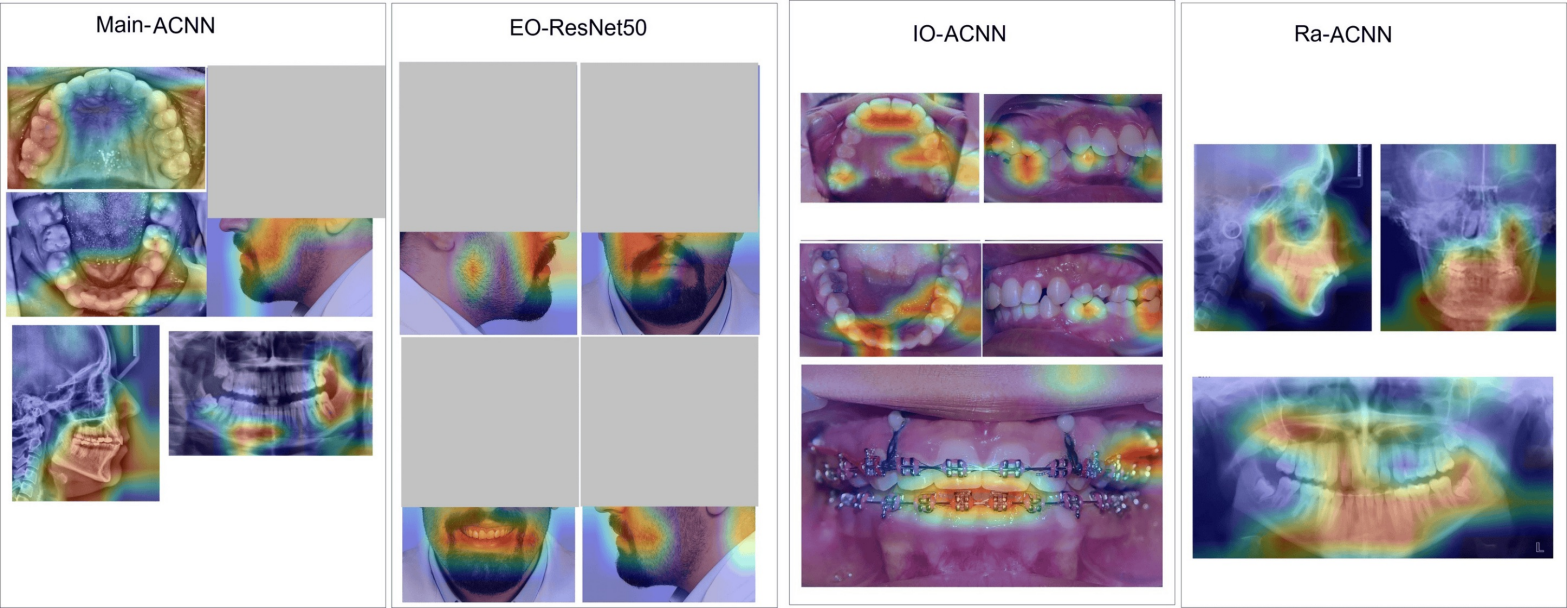
Grad-CAM visualizations for selected test data of each model. Warmer regions (e.g., red and yellow) highlight areas of the image that contribute more strongly to the model's prediction, while cooler regions (e.g., blue) indicate areas with less influence. Grad-CAM is a technique used to visualize the parts of the image that have the most impact on the model’s prediction. Grad-CAM: gradient-weighted class activation mapping; ACNN: attention-based convolutional neural network; IO-ACNN: intraoral model; EO-ACNN: extraoral model; Ra-ACNN: radiographic model

Figures 6, 7, 8, 9 illustrate the learning curves for the main, extraoral, intraoral, and radiographic models, respectively. Figures 10, 11, 12, 13 illustrate the confusion matrices for the main, extraoral, intraoral, and radiographic models, respectively. Figure 14 and Figure 15 illustrate the model's performance using predictions and normalized probabilities. Blue points represent correctly predicted samples, while red points indicate misclassified ones. The x-axis represents the normalized maximum probability, which indicates the model's confidence in class predictions, while the y-axis shows the sample indices. The normalized maximum probability was calculated by dividing the highest predicted probability for a class by the sum of all class probabilities, providing a measure of the model's confidence in predicting a specific class. The dashed black line represents the mean confidence of the predictions. Figure 16 illustrates the ROC curves for the best models. Figure 17 presents Grad-CAM visualizations for several test samples from each model, further improving the interpretability of their predictions.

**Figure 18:**
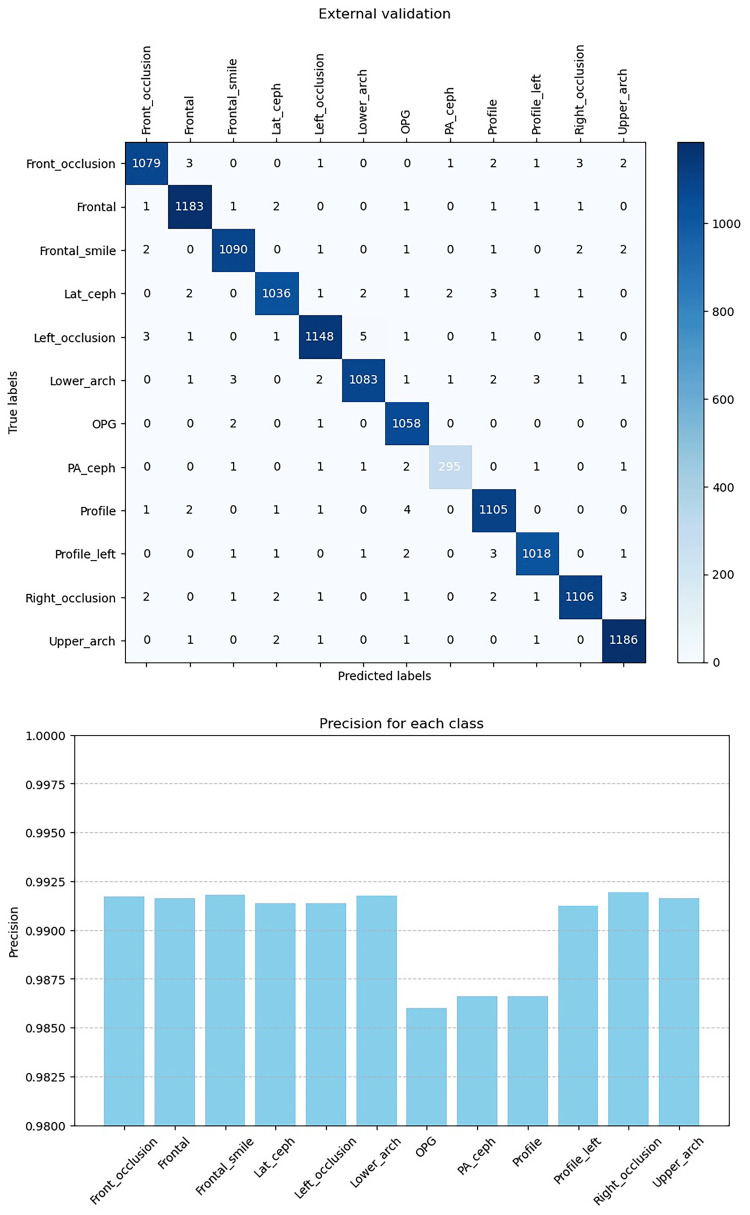
Performance assessment through external validation. The confusion matrix illustrates the performance of the final system after external validation by comparing the AI results with expert assessments. In this matrix, the software's classifications are considered as predicted labels, whereas the expert classifications represent the true labels. The diagonal entries represent correct classifications where the AI and expert agree, while off-diagonal entries indicate misclassifications, highlighting cases where the AI's predictions differ from the expert's judgment. This comparison helps assess the reliability of the AI system in replicating expert-level performance and identifying potential areas for improvement. Lat-Ceph: lateral cephalometry; OPG: orthopantomograph; PA-Ceph: posteroanterior cephalometry

Secondary tests revealed an average classification time of 8.02 seconds for the AI framework and 18.55 seconds for the orthodontist (Table 3). The Shapiro-Wilk test confirmed a non-normal distribution (p < 0.05) of classification times for both groups, and the Wilcoxon signed-rank test showed a significant time difference (p < 0.001), highlighting the efficiency of the AI framework. Both the orthodontist and the AI framework correctly classified all diagnostic images. The AI framework achieved an accuracy of 0.9924 in external validation on 13,729 new samples, with sensitivity ranging from 0.9672 to 0.9970 and specificity ranging from 0.9987 to 0.9997. Figure 18 displays the confusion matrix, comparing the AI's performance with that of the expert during external validation.

**Table 3:** Comparison of model (AI) and human (expert) diagnosis performance on 20 new clinical data (secondary test). AI: artificial intelligence

	Time Taken (seconds)		Expert Predictions		AI Predictions	
	Expert	AI	True	False	True	False
1	21.3	8.8	11	0	11	0
2	23.3	7.7	11	0	11	0
3	21.9	7.9	10	0	10	0
4	13.8	8.7	12	0	12	0
5	16.2	7.3	10	0	10	0
6	13.1	8.7	11	0	11	0
7	24.9	7.9	11	0	11	0
8	13.6	7.2	12	0	12	0
9	18.3	7.3	12	0	12	0
10	13.4	7.2	11	0	11	0
11	15.5	8.6	10	0	10	0
12	16.5	9.1	11	0	11	0
13	17.1	7.6	13	0	13	0
14	24.4	7.9	12	0	12	0
15	24.7	7.3	10	0	10	0
16	17.5	8.7	10	0	10	0
17	21.1	8.3	10	0	10	0
18	23.2	7.7	12	0	12	0
19	16.3	7.5	13	0	13	0
20	15	9.1	12	0	12	0

## Discussion

This study presents a deep learning method for automating the classification and archiving of orthodontic images using a sequential ACNN architecture, achieving superior accuracy compared to existing methods [26-28]. The model misclassified only one image out of 394 in a test set, resulting in an accuracy of 99.74%. External validation on a larger dataset of 13,729 images yielded a misclassification rate of 0.0093%, with 128 images misclassified. We employed a multi-stage classification strategy, developing four distinct models to address challenges encountered by a single model, particularly in distinguishing between frontal views at rest and frontal views with smiling expressions [27]. The original 13 classes were divided into three groups: extraoral, intraoral, and radiographic, allowing for the creation of specialized models for each group. This process begins with an initial model to determine the group, followed by a specialized model for final classification, all fully automated. In benchmarking our ACNN against ResNet50 and InceptionV3 [29], all three architectures achieved high accuracy (averaging 98.67% across four tasks), with ACNN outperforming the others in the main, intraoral, and radiographic tasks, while ResNet50 excelled in extraoral classification. The performance of both ResNet50 and ACNN is attributed to their deep convolutional architectures that effectively extract complex image features. ResNet50's residual learning framework allows for improved optimization and accuracy, while ACNN's attention modules enhance focus on relevant details, such as teeth. Overall, ResNet50 showed better performance in extraoral classification, whereas ACNN outperformed in other areas, although its confidence in radiographic classification was lower, likely due to insufficient training data or the complexity of radiographs.

To assess the clinical relevance of our model, we compared its performance in classifying orthodontic images with that of a human expert. The model achieved perfect accuracy, matching the expert's performance, while also demonstrating faster processing speeds, highlighting AI's potential to enhance clinical efficiency. This finding aligns with previous research [26], supporting the integration of AI into clinical workflows. Additionally, Grad-CAM visualizations confirmed that the model focuses on key features relevant to each image category, consistent with human judgment.

External validation with a new dataset from a different dental clinic provides insights into the model's real-world performance. Despite an impressive mean accuracy of 99.24%, lower precision was observed in the PA-ceph, OPG, and profile classes. Misclassifications may originate from the main or sub-models and could be attributed to image quality issues, such as low contrast and noise in PA-ceph and OPG images, or artifacts like braces and fillings that obscure key features. Variations in head positioning and subtle differences in facial features further complicate classification, especially after resizing to 128x128 pixels. Our sequential model achieved perfect accuracy (1.00 AUC) across intraoral, extraoral, and main models, with mean confidence scores approaching 0.99. However, the radiographic sub-model, achieving 99.24% accuracy and a confidence score of 0.96, struggled to distinguish OPG from Lat-ceph images, leading to the only misclassification in the test set. Grad-CAM visualizations indicate that the model may focus on overlapping anatomical features, such as teeth and the jawline, instead of more discriminative areas like the overall skull structure.

This study contributes to the existing literature by introducing ACNN, a customized deep learning architecture that incorporates attention modules. This design enables the model to learn specific features that capture subtle variations, particularly in distinguishing between smiling and non-smiling expressions and between front occlusion and side occlusion. This approach addresses the limitations of simpler CNN architectures and single-model methods used in previous studies [27]. While pre-trained models like DeepID [26], SqueezeNet [28], and ResNet50 have shown high performance in simpler tasks, our custom architecture with attention modules may offer advantages in more complex scenarios, such as analyzing malocclusion types or dental crowding severity. Additionally, unlike previous studies [26-28], our multi-stage approach allows for the selection of different sub-models based on image type, enhancing flexibility and robustness across various tasks. Finally, this study employs a significantly larger and more diverse dataset, offering greater robustness to variations in patient populations and demographics compared to previous research.

Comparing our model's results with previous studies shows promising advancements. While Li et al. [26] reported a high overall accuracy of 99.4%, their model has two key limitations: it relies solely on grayscale radiographic images, limiting applicability in poorly illuminated or cropped scenarios, and its reliance on aspect ratio for distinguishing between OPG and Lat-ceph images undermines reliability due to potential machine-dependent variations. Ryu et al. [27] achieved 98% accuracy on non-radiographic images from a test set of 100 samples, but their performance reached 100% accuracy in only three out of nine categories. They struggled particularly with distinguishing "frontal at rest" and "frontal at smile." In contrast, our model achieved perfect accuracy across all intraoral and extraoral categories using ACNN and ResNet50 architectures, respectively. The Grad-CAM visualizations of the extraoral model in our study highlight its ability to effectively distinguish between "frontal at rest" and "frontal at smile" images. Wang et al. [28] reported an overall accuracy of 99.84% using an enhanced SqueezeNet model, seemingly surpassing our model's 99.74%. However, a comprehensive comparison is limited due to the lack of accessible performance metrics in their study, published in a Chinese-language journal. Although employing different methodologies, these studies collectively emphasize the potential of CNNs for achieving high-accuracy orthodontic image classification. They underscore the capability of AI to address the growing need for automated indexing, comparison, and analysis of orthodontic images in contemporary digital practice.

This study has several limitations. First, it focused on a specific set of image categories, which may not fully represent the variety of images encountered in clinical practice, such as other radiographic images. However, the model's modular design allows for adaptation to different imaging scenarios and regional variations. Second, while manual annotation ensured accuracy, it was labor-intensive and time-consuming. Future research should explore automated annotation techniques to improve efficiency and scalability. Lastly, the study did not evaluate the models' computational efficiency or resource requirements, which are essential in resource-limited settings. Specifically, evaluating the hardware resources (such as processor, memory, and graphics processing unit (GPU) power) and software requirements (including software dependencies and operating systems) for running the AI framework would help researchers assess its applicability and usability in environments with limited resources. This is particularly important in countries or institutions with limited access to advanced computational resources. Addressing these limitations in future work could enhance the robustness and applicability of orthodontic image recognition systems in diverse clinical environments.

## Conclusions

In this study, we developed a deep learning framework for the automated classification and archiving of orthodontic records, including intraoral, extraoral, and radiographic images, categorized into 13 classes. The architecture, which integrates a multi-model classification approach and attention modules, enhances the model’s adaptability and accuracy in real-world applications. During external validation on a dataset of 13,729 images, the model achieved a mean accuracy of 99.23%. Slightly reduced performance was noted in the PA-ceph, OPG, and profile categories. Grad-CAM visualizations demonstrated that the model focused on clinically significant areas, aligning with expert diagnostic reasoning. This research represents a significant step forward in automating orthodontic diagnostic and treatment workflows, demonstrating the potential of CNNs for efficient classification and archiving. Ultimately, it helps reduce the workload of healthcare professionals and sets a strong foundation for future advancements in automated orthodontic systems and digital dentistry.
